# Ectopic Adrenocorticotropic Hormone Syndrome Secondary to Pulmonary Neuroendocrine Tumor: Medical Stabilization Enables Serial Imaging and Localization

**DOI:** 10.1016/j.aed.2025.10.016

**Published:** 2025-10-25

**Authors:** Muhammed Kizilgul, Ilitch Diaz-Gutierrez, Diana Oramas Mogrovejo, Ammar Ahmed, Lynn A. Burmeister, Kidmealem Zekarias

**Affiliations:** 1 University of Minnesota, Division of Diabetes, Endocrinology & Metabolism, Department of Medicine, Minneapolis, Minnesota; 2 University of Health Sciences, Etlik City Hospital, Department of Endocrinology and Metabolism, Ankara, Turkey; 3 University of Minnesota, Division of Thoracic and Foregut Surgery, Department of Surgery, Minneapolis, Minnesota; 4 University of Minnesota, Department of Laboratory Medicine and Pathology, Minneapolis, Minnesota

**Keywords:** Cushing's syndrome, DOTATATE PET, ectopic ACTH syndrome, neuroendocrine tumor, occult tumors

## Abstract

**Background:**

Ectopic adrenocorticotropic hormone (ACTH) syndrome accounts for 15% to 20% of Cushing syndrome cases with unique diagnostic challenges. Tumor localization remains difficult, with approximately 20% of cases having occult sources despite extensive imaging. This report describes a patient whose initially occult tumor was successfully localized through serial imaging enabled by medical stabilization, resulting in curative surgical resection.

**Case Presentation:**

Thirty-nine-year-old woman presented with progressive weight gain, new-onset hypertension, hypokalemia, proximal muscle weakness, and cushingoid features. Laboratory evaluation demonstrated severe hypercortisolism with markedly elevated ACTH levels, and inferior petrosal sinus sampling confirmed the diagnosis of ectopic ACTH syndrome. Despite comprehensive imaging—including cross-sectional studies, gallium-68 (68Ga)-DOTA-D-Phe1,Tyr3-octreotate positron emission tomography/computed tomography, and FDG PET/CT—the ectopic source remained elusive. Medical therapy with ketoconazole and metyrapone achieved rapid biochemical control. An 8 mm lingular pulmonary nodule, non-avid on both DOTATATE and FDG PET but identified on the CT portion of FDG PET/CT, was surgically resected, resulting in complete biochemical cure.

**Discussion:**

This case highlights medical stabilization's critical role when tumor localization is initially unsuccessful, enabling serial anatomic imaging that identified an 8 mm pulmonary carcinoid initially obscured by atelectasis and nonavid on functional imaging. Small, well-differentiated neuroendocrine tumors can cause severe hypercortisolism, yet remain undetectable on DOTATATE and FDG PET.

**Conclusion:**

This case demonstrates that medical stabilization achieves rapid biochemical control, providing time for serial anatomic imaging to localize occult ectopic ACTH sources. A small pulmonary carcinoid initially obscured by atelectasis and non:avid on functional imaging was identified through repeat CT comparison, enabling curative resection and avoiding bilateral adrenalectomy.

**Highlights**

• Medical stabilization enables serial imaging in occult ectopic adrenocorticotropic hormone (ACTH) syndrome
• Serial anatomic CT may reveal lesions missed on initial studies
• Functional imaging may miss small well-differentiated neuroendocrine tumors
• Bilateral adrenal uptake may represent ACTH-driven hyperplasia
• Small neuroendocrine tumors can cause life-threatening hypercortisolism

**Clinical Relevance**

Medical stabilization provides the critical time window for serial imaging studies when ectopic adrenocorticotropic hormone source is occult. Systematic clinical reasoning combined with repeat anatomic imaging can localize small hormonally-active tumors missed by functional imaging, enabling curative resection while avoiding bilateral adrenalectomy and lifelong adrenal insufficiency.

## Introduction

Ectopic adrenocorticotropic (ACTH) syndrome represents a challenging subset of Cushing syndrome, characterized by ACTH secretion from non-pituitary sources.^1^ The clinical presentation can be severe, with rapid onset of symptoms due to markedly elevated cortisol levels.^1^^,^^2^ Common sources include lung tumors, pancreatic neuroendocrine and thymic tumors.

The greatest challenge is accurate tumor localization. Despite advances in imaging including CT, MRI, and functional imaging with gallium-68 (68Ga)-DOTA-D-Phe1,Tyr3-octreotate (68Ga-DOTATATE) PET or 18F-fluorodeoxyglucose PET/CT, some cases remain occult after exhaustive imaging.^1^ Medical therapy with adrenal steroidogenesis inhibitors controls hypercortisolism while allowing time for comprehensive tumor localization and avoiding bilateral adrenalectomy.^2^, ^3^, ^4^, ^5^, ^6^ Here we present a case of ectopic ACTH whose initially occult tumor source was successfully localized through serial imaging enabled by medical stabilization therapy.

## Case Presentation

A 39-year-old woman with a past medical history of post-traumatic stress disorder presented with a 1-year history of progressive cushingoid symptoms, including a 40-pound weight gain, new-onset hypertension, hypokalemia, new-onset prediabetes, easy bruising, proximal muscle weakness, mental cloudiness, fatigue, bilateral lower extremity edema, menstrual irregularity and progressive acne with hair loss. Physical examination revealed hypertension (159/124 mmHg), obesity (body mass index 32.7 kg/m^2^), and classic cushingoid features, including central obesity with buffalo hump, facial plethora and moon facies, purple abdominal striae, acneiform lesions on the torso, proximal muscle weakness, and bilateral lower extremity edema.

Initial biochemical testing demonstrated severe hypercortisolism. Morning serum cortisol levels were markedly elevated at 33.9-40.4 μg/dL (reference: 6.2–19.4 μg/dL). A 24-hour urinary free cortisol was profoundly increased at 2484 μg/24h (reference: ≤45 μg/24h). Low-dose (1 mg) dexamethasone suppression testing failed to suppress cortisol (39.8 μg/dL; reference: <1.8 μg/dL), and high-dose (8 mg) testing similarly failed, with cortisol rising further to 50.6 μg/dL. Baseline plasma ACTH was markedly elevated at 125 pg/mL (reference: <47 pg/mL), consistent with ACTH-dependent Cushing syndrome. A desmopressin stimulation test demonstrated a modest rise in ACTH from 84 pg/mL to 156 pg/mL (30% increase), raising the possibility of a pituitary source. However, inferior petrosal sinus sampling showed baseline central-to-peripheral ACTH ratios of 1.12 (right) and 1.30 (left), and post-stimulation ratios of 1.42 (right) and 1.56 (left)—all below diagnostic thresholds (>2.0 at baseline or >3.0 after stimulation)—excluding a pituitary source and confirming ectopic ACTH syndrome. Additional workup revealed severe hypokalemia, with a nadir potassium of 2.1 mEq/L (reference: 3.5–5.0 mEq/L) requiring intensive repletion, metabolic alkalosis (CO2 33 mmol/L; reference: 22-29 mmol/L), and new-onset hyperglycemia (fasting glucose 111 mg/dL; reference: 70-99 mg/dL) with hemoglobin A1c of 6.2% (reference: <5.7%). Screening for other potential endocrine tumors, including 24-hour urinary 5-hydroxyindoleacetic acid, plasma metanephrines, and calcitonin, were within normal limits.

Pituitary MRI with contrast showed partially empty sella with no adenoma. Initial CT of the chest, abdomen, and pelvis revealed an ill-defined 1.3 × 0.8 cm left adrenal nodule and minimal streaky atelectasis within the lingula and right middle lobe. Abdominal MRI confirmed mild bilateral adrenal thickening and an 8 mm left adrenal nodule with imaging features consistent with a lipid-rich adenoma. Functional imaging with 68Ga-DOTATATE PET/CT demonstrated asymmetric focal uptake in the left adrenal gland (standardized uptake value [SUV] max 17.3, Krenning score 3) compared to the right adrenal gland (SUV max 9.0). As the source of ACTH secretion remained inconclusive and the patient had severe hypercortisolism, medical therapy with adrenal steroidogenesis inhibitors was initiated for disease control. Follow-up 18F-fluorodeoxyglucose PET/CT 1 month later revealed symmetric mild uptake in both adrenals (SUV max 5.3 left, 4.8 right), while the CT portion identified non–FDG-avid 8 mm lingular pulmonary nodule. The nodule was initially overlooked due to atelectasis. Medical management with ketoconazole (initiated at 200 mg twice daily, titrated to 200 mg 3 times daily) and metyrapone (initiated at 500 mg 3 times daily, titrated to 750 mg 3 times daily) was well-tolerated without hepatotoxicity or other adverse effects. This therapy led to rapid improvement, with 24-hour urine cortisol decreasing from >2000 μg/24h to 357 μg/24h within 2–3 weeks accompanied by marked improvement in blood pressure control. Initial antihypertensive therapy included amlodipine 10 mg daily, lisinopril 40 mg daily, hydrochlorothiazide 25 mg daily, spironolactone 200 mg daily, and carvedilol 12.5 mg twice daily, which was reduced to only spironolactone 100 mg daily and amlodipine 10 mg daily following cortisol normalization.

**Fig. 1:**
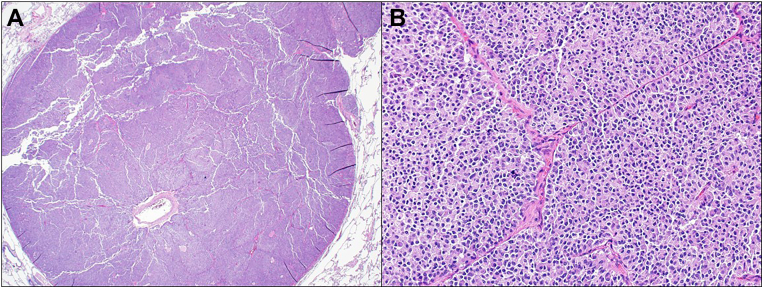
Hematoxylin and eosin stain, A. 1x view shows a well circumscribed nodule with epithelioid cells without necrosis. B. 20x view shows epithelioid cells with abundant eosinophilic cytoplasm and prominent nuclei with salt and pepper chromatin. Intersecting vessels are seen. Mitotic activity is low.

**Fig. 2:**
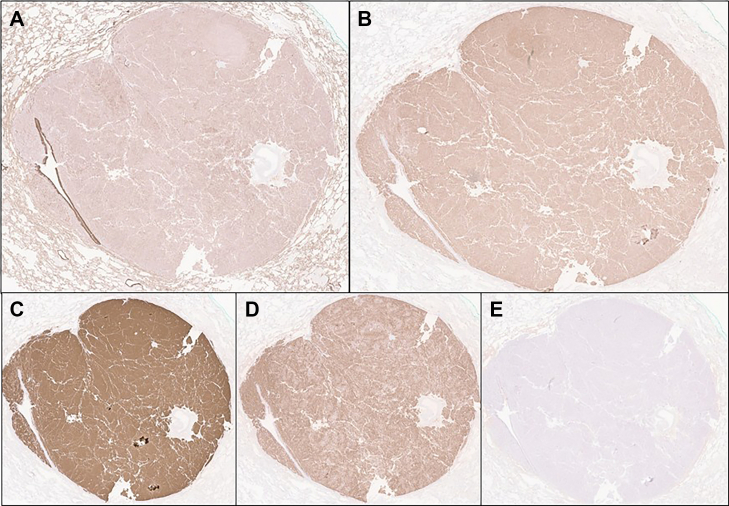
Immunohistochemical stains, A shows cytokeratin AE1/AE3 positive in the tumor cells. B. ACTH stain is diffusely positive. C&D. Synaptophysin and chromogranin, respectively, are diffusely and strongly positive. E. Ki67 proliferative index is 1%.

Within 2 weeks of localizing the pulmonary nodule, the patient underwent a left upper lobe wedge resection with lymph node sampling targeting the 8 mm lingular lesion. Gross examination confirmed a 1 cm pulmonary nodule, and microscopic evaluation revealed epithelioid cells with abundant eosinophilic cytoplasm, prominent nuclei with salt-and-pepper chromatin, and an intervening vascular network. No necrosis was identified, and mitotic activity was low (<1 mitosis/2 mm^2^). Immunohistochemical staining demonstrated positivity for the epithelial marker cytokeratin AE1/AE3, as well as diffuse strong positivity for the neuroendocrine markers synaptophysin and chromogranin A. ACTH staining was also diffusely positive, confirming the lesion as the source of ectopic hormone secretion. The Ki-67 proliferative index was 1% (Figs. 1 and 2). The final diagnosis was a typical ACTH-producing pulmonary carcinoid tumor, measuring 1.0 cm, with negative margins, no visceral pleural invasion, and no lymph node involvement (0/2 in stations 5 and 7). Pathologic staging was pT1a, *N*0 according to AJCC criteria. The postoperative course was uncomplicated, and biochemical testing revealed undetectable ACTH levels (<10 pg/mL) and morning cortisol (1.4 μg/dL at 8:00 am), confirming complete biochemical cure of the ectopic ACTH syndrome. Postoperatively, the patient experienced adrenal insufficiency requiring hydrocortisone replacement (15 mg morning, 7.5 mg afternoon). Blood pressure normalized immediately, allowing discontinuation of all antihypertensives. Metabolic parameters rapidly improved with normalization of glucose and resolution of hypokalemia.

## Discussion

This case demonstrates the diagnostic challenges of localizing ectopic ACTH sources despite contemporary imaging modalities.^7^

The desmopressin test showed a 30% ACTH rise, an unexpected finding in ectopic ACTH syndrome. This likely represented a false-positive response due to V2 vasopressin receptor expression in ectopic tumors,^8^, ^9^, ^10^ which can mimic pituitary responsiveness and necessitated inferior petrosal sinus sampling for definitive source localization.^3^

**Fig. 3:**
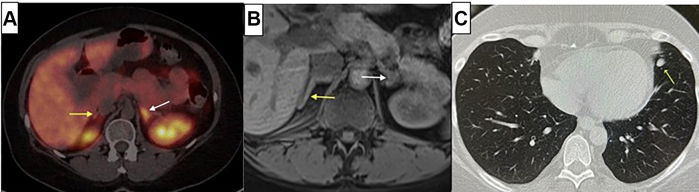
Multimodality imaging findings: (A) ^68^Ga-DOTATATE PET/CT revealed asymmetric focal radiotracer uptake in the *left* adrenal gland (standardized uptake value [SUV]max 17.3, Krenning score 3) (white arrow) compared to the *right* adrenal gland (SUVmax 9.0) (yellow arrow). (B) Abdominal magnetic resonance imaging demonstrated mild bilateral adrenal thickening (yellow and white arrows) with an 8 mm *left* adrenal nodule (white arrow) showing imaging characteristics consistent with a benign lipid-rich adenoma. (C) Chest CT from follow-up 18F-fluorodeoxyglucose PET/CT study identified an 8 mm lingular pulmonary nodule (white arrow).

DOTATATE PET/CT demonstrated asymmetric left adrenal uptake (SUV max 17.3 vs 9.0), raising initial suspicion for ACTH production from an adrenal source (Fig. 3*A*). While pheochromocytomas can rarely produce ectopic ACTH,^11^ this possibility was effectively excluded by normal plasma metanephrine and normetanephrine levels, along with MRI features indicative of a benign lipid-rich adenoma^12^ (Fig. 3*B*). Subsequently, the symmetric FDG uptake (SUV max 5.3 left, 4.8 right) was consistent with hyperplasia rather than a functioning adrenal tumor, which typically shows asymmetric intense uptake.^1^^,^^2^^,^^13^

Our initial imaging strategy prioritized 68Ga-DOTATATE PET/CT, which demonstrates higher sensitivity (75%) compared to FDG PET/CT (60%) for detecting ectopic ACTH-secreting tumors, with combined modalities achieving 90% detection rates.^13^ When DOTATATE showed no definitive pulmonary lesion, complementary 18F-fluorodeoxyglucose PET/CT was pursued as part of our comprehensive workup enabled by medical stabilization. Notably, the 8 mm lingular nodule was non-avid on both DOTATATE and FDG imaging but was identified on the CT portion of the FDG PET/CT study (Fig. 3*C*), likely because repeat imaging allowed comparison with prior studies and the atelectasis had resolved.

The absence of functional imaging avidity likely reflects the tumor’s small size (8 mm), well-differentiated histology (Ki-67 1%), and possible suppression of somatostatin receptor subtype 2 expression by severe hypercortisolism.^13^^,^^14^ Small, well-differentiated neuroendocrine tumors may remain non-avid on both DOTATATE and FDG PET despite hormonal activity. This case underscores that anatomic cross-sectional imaging on serial studies remains critical even when functional imaging is negative.^2^^,^^13^

Despite the lack of functional imaging avidity, surgical resection was pursued given the nodule's anatomic characteristics, the exclusion of alternative sources, and pulmonary carcinoids being the most common cause of ectopic ACTH syndrome.^1^^,^^7^ The alternative (bilateral adrenalectomy) would have caused lifelong adrenal insufficiency without addressing the tumor.

Medical therapy with ketoconazole and metyrapone achieved rapid biochemical control, preventing complications and providing the critical window needed for serial anatomic imaging that ultimately localized the tumor, thereby avoiding bilateral adrenalectomy.^2^^,^^4^^,^^6^

Surgical resection of the identified 8 mm lingular pulmonary carcinoid achieved complete biochemical cure, with undetectable ACTH levels and appropriately suppressed morning cortisol. Histopathology confirmed a well-differentiated, ACTH-producing typical carcinoid (Ki-67 1%, pT1a, *N*0) with favorable prognosis. The immediate normalization of blood pressure, resolution of metabolic abnormalities, and development of adrenal insufficiency requiring replacement therapy validated the tumor as the sole source of ACTH secretion and supports the stepwise diagnostic and therapeutic approach employed in this case.

## Conclusion

This case demonstrates that medical stabilization enables curative surgical treatment in occult ectopic ACTH syndrome when initial imaging is unrevealing. Key clinical lessons: (1) Medical therapy provides the critical time window for serial imaging when the ectopic source is occult, avoiding urgent bilateral adrenalectomy; (2) Repeat anatomic CT comparison can identify small lesions initially obscured by atelectasis or other processes; (3) Small, well-differentiated neuroendocrine tumors causing severe hypercortisolism may remain nonavid on both DOTATATE and FDG PET. Clinicians managing occult ectopic ACTH should prioritize medical stabilization and pursue serial anatomic imaging even when functional studies are negative, as systematic comparison of repeat cross-sectional imaging may reveal previously overlooked tumors.

## Declaration of Generative AI and AI-Assisted Technologies in the Writing Process

During the preparation of this work, the author(s) used claude.ai in order to improve readability and language. After using this tool, the author(s) reviewed and edited the content as needed and take(s) full responsibility for the content of the publication.

## Disclosure

The authors have no conflicts of interest to disclose.
